# Benefits of switching from guaiac-based faecal occult blood to faecal immunochemical testing: experience from the Wallonia–Brussels colorectal cancer screening programme

**DOI:** 10.1038/s41416-020-0754-5

**Published:** 2020-02-18

**Authors:** Feng Guo, Isabel De Brabander, Julie Francart, Michel Candeur, Marc Polus, Liesbet Van Eycken, Hermann Brenner

**Affiliations:** 1 Division of Clinical Epidemiology and Aging Research, German Cancer Research Center (DKFZ), Heidelberg, Germany; 2 Medical Faculty Heidelberg, University of Heidelberg, Heidelberg, Germany; 3 Belgian Cancer Registry (BCR), Brussels, Belgium; 4 Community Reference Center for Cancer Screening (Wallonia), Mont-Saint-Guibert, Belgium; 5 Department of Gastroenterology, University Hospital of Liège, Liège, Belgium; 6 Division of Preventive Oncology, German Cancer Research Center (DKFZ) and National Center for Tumor Diseases (NCT), Heidelberg, Germany; 7 German Cancer Consortium (DKTK), German Cancer Research Center (DKFZ), Heidelberg, Germany

**Keywords:** Health policy, Cancer screening, Cancer epidemiology

## Abstract

**Background:**

Faecal immunochemical tests (FITs) have replaced guaiac-based faecal occult blood test (gFOBTs) in several colorectal cancer (CRC) screening programmes. We aimed to evaluate the benefits of this transition based on the Wallonia–Brussels-organised CRC screening programme.

**Methods:**

A total of 1,569,868 individuals aged 50–74 years, who were invited to screening during 2009–2017, were studied by linking their screening records with insurance, pathology and cancer data in the Belgian Cancer Registry. We compared neoplasm detection rates and positive predictive values (PPVs) of gFOBT and FIT at 15 µg haemoglobin per gram cut-off in screen-naive individuals. We furthermore examined the incidence rates of interval cancer in gFOBT- and FIT-based screening programme.

**Results:**

Advanced neoplasms were detected less frequently by gFOBT (0.8%) than by FIT (1.3%), with a difference of 0.5% (*P* < 0.01). PPVs were lower for gFOBT (15.1%) than for FIT (21.7%) for advanced neoplasms (difference 6.6%, *P* < 0.01). Compared to participants with negative gFOBT, those with negative FIT were 77% less likely to develop interval cancer (incidence rate ratio 0.23, 95% confidence interval 0.16–0.33).

**Conclusion:**

Our study demonstrated that in an organised CRC screening programme, replacing gFOBT with FIT improved neoplasm detection rate and substantially reduced interval cancer incidence.

## Background

Colorectal cancer (CRC) is the third most commonly diagnosed cancer and the second leading cause of cancer-related mortality worldwide.^1^ A substantial proportion of CRC deaths can be prevented by screening. Over many years, the most commonly used non-invasive test for CRC screening has been the guaiac-based faecal occult blood test (gFOBT). Offering annual gFOBT screening has been shown to reduce CRC mortality by up to 30% in randomised clinical trial, although the sensitivity of this test to detect CRC and its precursors has been very limited.^2,3^

In recent years, faecal immunochemical tests (FITs), which were shown to be more sensitive for colorectal adenomas and CRCs,^4–9^ have replaced gFOBT or have been newly introduced in many CRC screening programmes.^8,10^ However, according to a recent European Union-funded report on cancer screening programmes, in some countries, such as Croatia, Finland, Latvia and Sweden, gFOBTs were still offered as primary CRC screening tests.^11^ Previous studies have demonstrated that replacing gFOBT by FIT in pilot screening programmes markedly increased the participation rate and yield of advanced neoplasia.^12–14^ Although implementation of FIT-based screening has been widely recommended, empirical evidence on the impact of switching from gFOBT- to FIT-based screening on key outcome variables of screening on the population level is yet very limited.

In the Walloon and Brussels-capital region of Belgium, an organised gFOBT-based CRC screening programme had been fully implemented since March 2009 and transited to FIT-based screening in March 2016. In this study, we aimed to evaluate the neoplasm detection rates, positive predictive values (PPVs), characteristics of screen- and non-screen-detected cancers and incidence rates of interval cancer in the Wallonia–Brussels-organised CRC screening programme before and after this transition.

## Methods

### Wallonia–Brussels CRC screening programme

Since March 2009, an organised gFOBT-based screening programme has been fully implemented in Wallonia and Brussels. The screening programme covers the population from the French- and German-speaking community of Belgium. Women and men aged 50–74 years were invited biennially and asked to collect a test kit for the purpose of CRC screening from their general practitioners (GPs), together with a prepaid envelope for returning the completed test kit. In March 2016, the Wallonia–Brussels CRC screening guideline was officially updated by replacing gFOBT with FIT. For both the gFOBT- and FIT-based screening programme, no specific reminders were sent to persons who had not collected a test kit from their GPs or had not returned a completed test after receiving a test kit.

The gFOBT used in Wallonia and Brussels was Hemoccult II (Beckman Coulter). For FIT, an automated quantitative FIT (OC-Sensor, Eiken Chemical, Tokyo, Japan) was offered. The analyses of returned gFOBT and FIT samples were done at the Walloon Central Laboratory (Mont-Saint-Guibert, Belgium). Positivity of gFOBT was defined as blue discoloration of any of the six returned stool samples (on three cards) within 30–60 s after applying the developing solution. The positivity threshold for FIT was set at 15 µg haemoglobin per gram (Hb/g) faeces. Detailed information on sample collection and handling, laboratory analysis, quality management and data handling according to the checklist for FITs for Hb evaluation reporting (FITTER)^15^ are provided in the Supplementary Appendix.

The GPs of the screening participants were informed on the test result within 5 days after analysis. Participants with negative result were suggested to attend screening again after 2 years. A major change regarding subsequent screening invitation was made in March 2015. Before then, participants with prior negative result were invited again by a letter without inclusion of a faecal test kit, whereas an invitation letter along with a test kit was sent to them from March 2015 on. Participants with a positive result were advised to undergo colonoscopy. Colonoscopies for follow-up of positive gFOBT/FIT results were conducted by gastroenterologists, specialists in internal medicine, or surgeons in hospitals or private practices.^16^

The faecal test (either gFOBT or FIT) was offered free of charge for all individuals invited for screening, while the advised follow-up colonoscopy, medical care and treatment were partially reimbursed by the health insurance company. The proportion of reimbursement varied according to personal circumstances (health status, employment status, financial status, etc.).

### Databases for this study

The data used in this study included individuals aged 50–74 years who were invited for CRC screening between March 2009 and June 2017. Overall, 1,569,868 individuals (195,170 persons screened at least once, and 1,374,698 persons never screened) were included in our analysis. Information on screening invitation, participation, type of faecal test and test results were obtained from the Walloon screening organisation. Although FIT has been officially offered instead of gFOBT since March 2016 in Wallonia and Brussels, due to inventory management many clinical settings continued offering surplus gFOBT kits until June 2017. Additionally, a few GPs have provided FIT to attendees since January 2014 on the background of a pilot study, which aimed to adapt to the changes in switching screening tests. The present study therefore includes gFOBTs and FITs, which have been offered between March 2009 and June 2017, and between January 2014 and June 2017, respectively.

Data on adherence to, date and completeness of follow-up colonoscopy were obtained from the Intermutualistic Agency (IMA) database, which covers nearly 99% of the Belgian population and includes data collected in the framework of the compulsory Belgian health care and benefits insurance programme, on reimbursed medication and the use of reimbursed health services (up to date until December 2017 by the time of this analysis).

After obtaining the information on use of screening test and follow-up examination, the study population was linked to the Central Cyto-Histopathology (CHP) and Cancer Registry (CR) databases in the Belgian CR (BCR). The BCR is engaged in the assessment of quality and effectiveness of the Belgian national organised screening programmes for colorectal, cervical and breast cancer. The CHP database documents the histopathological results of colorectal samples from individuals with or without screening and covers results from all pathological laboratories (pathology network) in Belgium. All data that enters the CHP database are submitted to an extended set of automated and manual validation procedures to ensure validity and quality of the data. The histopathologic information was used to validate CRC diagnosis. Approximately 99% of CRC cases registered at the BCR have been microscopically verified.^17^ Data on characteristics of tumour (location and stage) and patient (age and sex) for all identified CRC cases between March 2009 and December 2016 were collected from the CR database, and these characteristics (except for tumour stage) for CRCs diagnosed in 2017 were ascertained from the CHP database.

Morphology and topography of colorectal lesions were coded with the International Classification of Diseases for Oncology, 3rd version. Neoplasms were defined as proximal if located from the caecum to the splenic flexure and as distal otherwise. Tumour stage was classified according to the TNM (tumour node metastasis) 6th (year of diagnosis: 2009) or 7th (year of diagnosis: 2010–2017) edition. In our study, advanced adenomas were defined as adenomas with tubulovillous or villous histology, or those with high-grade dysplasia. Patients with intramucosal carcinoma or carcinoma in situ were classified as having high-grade dysplasia. Cancers were defined by the invasion of malignant cells beyond the muscularis mucosa. Each patient was classified based on the most advanced lesion. An overview on the time periods covered by the various databases and used in our analysis is given in Supplementary Fig. 1.

### Screen- and non-screen-detected CRC

Screen-detected CRCs were defined as cancers diagnosed within 6 months of colonoscopy following a positive gFOBT or FIT result. Non-screen-detected CRCs were defined as CRCs that were not diagnosed within the screening programme and divided into four groups: (1) gFOBT/FIT interval cancers, defined as cancers diagnosed between screening rounds after negative gFOBT/FIT and before the next recommended test. (2) Colonoscopy interval cancers, defined as cases in which a follow-up colonoscopy was performed more than 6 months before CRC diagnosis. (3) CRCs in colonoscopy non-compliers, defined as cancers diagnosed in those who received a positive gFOBT/FIT result, but did not undergo a follow-up colonoscopy. (4) CRCs in non-attendees, defined as cancers diagnosed in those who did not collect a test kit from their GP or did not return the completed gFOBT/FIT kit. The rationale of using 6 months as a time window to distinguish screen-detected cancer and colonoscopy interval cancer is based on the assumption that CRCs suspected/detected at colonoscopy would be diagnosed within 6 months of the index procedure, and this definition has been previously used in several studies.^18–20^

### Statistical analysis

In the first set of analyses, we included screen-naive individuals who only attended one screening round to compare test positivity, colonoscopy adherence, detection rate and PPV of gFOBT with FIT. The comparison of participation rates in gFOBT and FIT screening was not included in the primary analysis, as the invitation letter did not specifically address the type of faecal test. We calculated the positivity rate as the number of persons with a positive gFOBT/FIT relative to the number of persons returning a gFOBT/FIT. The rate of colonoscopy adherence was calculated as the number of participants undergoing follow-up colonoscopy relative to the number of positive tests. The detection rate was calculated as the number of participants with lesions relative to the number of participants screened. The PPV was calculated as the number of detected lesions relative to the total number of follow-up colonoscopies. Furthermore, sex- and age-stratified detection rates for any advanced neoplasm (advanced adenoma or CRC) among gFOBT and FIT users were calculated. Rates and rate differences of above-mentioned outcomes were calculated, and all percentages were reported with 95% confidence interval (CI).

In the second set of analyses, we evaluated the characteristics of screen- and non-screen-detected CRCs as well as the incidence rates of interval cancer in gFOBT- and FIT-based screening programmes. The proportion of gFOBT/FIT interval cancers was calculated by dividing the number of gFOBT/FIT interval cancers by the sum of screen-detected and gFOBT/FIT interval cancers. Descriptive analysis was performed on CRC patient (sex, age) and tumour (location, stage) characteristics for gFOBT and FIT users separately. Differences in proportions between CRC groups were assessed using the *χ*^2^ test. Incidence rates of gFOBT and FIT interval cancer were calculated per 10,000 person-years. The follow-up time period was calculated as the time between negative test and interval cancer occurrence or censoring (either by December 2017 or by 2-year follow-up), whichever came first. An incidence rate ratio (IRR) and the corresponding 95% CI were calculated to compare the incidence rates of gFOBT and FIT interval cancers.

To fully reflect the number of advanced adenomas and CRCs that have been detected in the gFOBT- or FIT-based screening programme, compliance to follow-up colonoscopy was considered as colonoscopy conducted within 24 months after positive gFOBT/FIT. We additionally reported results on colonoscopy completion rate, detection rate, PPV, characteristics of screen-detected CRC and patients by considering follow-up colonoscopy as colonoscopy conducted within 6 months after positive faecal test.

All analyses were performed with SAS Enterprise Guide version 7.1 (SAS Institute, Cary, NC, USA). Two-sided *P* values <0.05 were considered statistically significant.

## Results

### Test positivity, detection rate and PPV

**Fig. 1:**
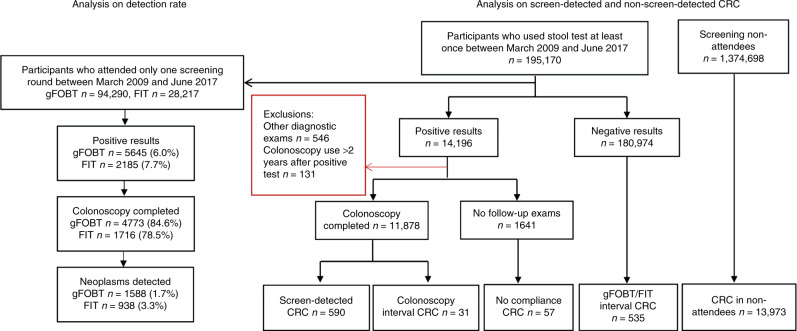
**Flow diagram for analyses on detection rate, screen- and non-screen-detected CRCs in the Wallonia–Brussels-organised gFOBT- and FIT-based screening programme.**

From 195,170 screening participants, 122,507 individuals attended only one screening round, among whom 94,290 used gFOBT and 28,217 used FIT (Fig. 1). The mean age was similar for gFOBT users (mean: 60.9 ± standard deviation: 7.3 years) and FIT users (59.9 ± 6.9 years). Of the gFOBT and FIT participants, 5645 (6.0%) and 2185 (7.7%) tested positive, resulting in a difference in positivity rate of 1.7% (95% CI: 1.4–2.1; Table 1). Follow-up colonoscopy completion rates were higher for gFOBT (84.6%) vs. FIT (78.5%), with a difference of −6.1% (95% CI: −8.0 to −4.0). Advanced neoplasms were found in 720 of the gFOBT participants (0.8%) and in 373 of the FIT participants (1.3%), with a difference of 0.5% (95% CI: 0.4–0.7). PPVs were also lower for gFOBT than for FIT: 6.5% vs. 6.9% for CRC (difference 0.4%, 95% CI: −1.0 to 1.8), and 15.1% vs. 21.7% for any advanced neoplasm (difference 6.6%, 95% CI: 4.5–8.9). The PPVs for above-mentioned outcomes remained almost unchanged when considering follow-up colonoscopy as colonoscopy conducted within 6 months after positive faecal test (Supplementary Table 1).

**Table 1:** Indicators of test performance of gFOBT vs. FIT.

	Type of stool test used for screening				Difference^a^
	gFOBT Total *N* = 94,290		FIT Total *N* = 28,217		
	*n*	% (95% CI)	*n*	% (95% CI)	% (95% CI)
Positive gFOBT/FIT results	5645	6.0 (5.8–6.1)	2185	7.7 (7.4–8.1)	1.7 (1.4–2.1)
Colonoscopy completion	4773	84.6 (83.6–85.5)	1716	78.5 (76.8–80.3)	−6.1 (−8.0 to −4.1)
Detection rate					
Colorectal cancer	310	0.3 (0.3–0.4)	119	0.4 (0.3–0.5)	0.1 (0.0–0.2)
Advanced adenoma	410	0.4 (0.4–0.5)	254	0.9 (0.8–1.0)	0.5 (0.3–0.6)
Any advanced neoplasm	720	0.8 (0.7–0.8)	373	1.3 (1.2–1.5)	0.5 (0.4–0.7)
Non-advanced adenoma	868	0.9 (0.9–1.0)	565	2.0 (1.8–2.2)	1.1 (0.9–1.3)
Any adenoma	1278	1.4 (1.3–1.4)	819	2.9 (2.7–3.1)	1.5 (1.3–1.8)
Positive predictive value^b^					
Colorectal cancer	310	6.5 (5.8–7.2)	119	6.9 (5.7–8.1)	0.4 (−1.0 to 1.8)
Any advanced neoplasm	720	15.1 (14.1–16.1)	373	21.7 (19.8–23.7)	6.6 (4.5–8.9)

*CI* confidence interval, *FIT* faecal immunochemical test, *gFOBT* guaiac-based faecal occult blood test.

^a^Differences with a 95% CI completely lower or higher than 0 are statistically significant, which means that the *P* value does not exceed 0.05.

^b^Positive predictive value is the percentage of participants with detected neoplasms relative to the number of participants with follow-up colonoscopy completed.

The detection rates were higher for men than for women in both gFOBT- and FIT-based screening programmes, but the detection rates were consistently higher in the latter screening programme (Table 2). No clear trend in detection rates with age was seen for gFOBT participants. In contrast, detection rates among participants screened with FIT rose across age categories, increasing from 0.8% at age 50–59 years to 2.3% at age ≥70 years for any advanced neoplasm.

**Table 2:** Detection rates of gFOBT and FIT for any advanced neoplasm, by sex and age.

	Type of stool test used for screening				Difference^a^
	gFOBT Total *N* = 94,290		FIT Total *N* = 28,217		
	*n*	% (95% CI)	*n*	% (95% CI)	% (95% CI)
Total	720	0.8 (0.7–0.8)	373	1.3 (1.2–1.5)	0.5 (0.4–0.7)
Sex					
Male	476	1.1 (1.0–1.2)	243	1.8 (1.6–2.0)	0.7 (0.4–0.9)
Female	244	0.5 (0.4–0.5)	130	0.9 (0.7–1.1)	0.4 (0.3–0.6)
Age					
50–59 years	235	0.5 (0.5–0.6)	127	0.8 (0.7–1.0)	0.3 (0.1–0.4)
60–69 years	355	1.0 (0.9–1.1)	184	1.8 (1.6–2.1)	0.8 (0.5–1.1)
≥70 years	130	0.8 (0.6–0.9)	62	2.3 (1.7–2.8)	1.5 (1.0–2.1)

*CI* confidence interval, *FIT* faecal immunochemical test, *gFOBT* guaiac-based faecal occult blood test.

^a^Differences with a 95% CI completely lower or higher than 0 are statistically significant, which means that the *P* value does not exceed 0.05.

### Proportion of screen- and non-screen-detected CRCs

During March 2009–June 2017, a total of 1,569,868 individuals were eligible for CRC screening, of whom 195,170 (12.4%) participated at least once (Fig. 1). Of these, 14,196 (7.3%) had a positive faecal test and 11,878 subsequently underwent colonoscopy (83.7% compliance). In the total population of 1,569,868, CRC was detected in 1213 screening participants and in 13,973 who never participated. The CRCs detected in the 1213 participants included 590 screen-detected CRCs (48.6%), 535 gFOBT/FIT interval cancers (44.1%), 31 colonoscopy interval cancers (2.6%) and 57 CRCs in colonoscopy non-compliers (4.7%). While the proportion of gFOBT interval cancer was 54.1% (503 interval cancers vs. 426 screen-detected CRCs), reflecting a gFOBT sensitivity for detecting CRC of ~46%, the proportion of FIT interval cancer was 16.3% (32 interval cancers vs. 164 screen-detected CRCs), reflecting a FIT sensitivity for detecting CRC of ~84%. When considering follow-up colonoscopy as colonoscopy conducted within 6 months after positive faecal test, the proportions of interval cancer were essentially unchanged (55.2% for gFOBT and 16.8% for FIT, Supplementary Table 2)

### Characteristics of CRC patients

Table 3 shows the characteristics of CRC patients within the screening programme. Screen-detected cancer, gFOBT/FIT interval cancer, colonoscopy interval cancer and CRC in non-attendees were found predominantly in men and among people aged 60–69 years in the overall screening programme; however, CRC cases among those who did not adhere to follow-up colonoscopy were quite equally distributed between sexes and across age groups. While significant differences were observed between patients with screen- and non-screen-detected CRCs for age (*P* = 0.013) and sex (*P* = 0.014) in the overall screening programme, there were only significant sex differences between the two groups of patients in gFOBT-based screening (*P* = 0.002) and only significant age differences in FIT-based screening (*P* = 0.003).

**Table 3:** Characteristics of CRC patients in the Wallonia–Brussels-organised gFOBT- and FIT-based screening programme.

	Screen-detected CRC	Non-screen-detected CRC					*P* value^b^
		Interval CRC		No colonoscopy compliance	CRC in non-attendees^a^	Overall	
		gFOBT/FIT	Colonoscopy				
gFOBT + FIT							
Total CRC	590	535	31	57	13 973	14,596	
Age, *n* (%)							
50–59 years	137 (23.2)	109 (20.4)	4 (12.9)	18 (31.6)	3672 (26.3)	3803 (26.0)	0.013
60–69 years	303 (51.4)	270 (50.5)	15 (48.4)	19 (33.3)	6291 (45.0)	6595 (45.2)	
≥70 years	150 (25.4)	156 (29.1)	12 (38.7)	20 (35.1)	4010 (28.7)	4198 (28.8)	
Sex, *n* (%)							
Male	370 (62.7)	287 (53.6)	22 (71.0)	28 (49.1)	8072 (57.8)	8409 (57.6)	0.014
Female	220 (37.3)	248 (46.4)	9 (29.0)	29 (50.9)	5901 (42.2)	6187 (42.4)	
gFOBT							
Total CRC	426	503	28	51	–	582	
Age, *n* (%)							
50–59 years	109 (25.6)	104 (20.7)	3 (10.7)	14 (27.4)	–	121 (20.8)	0.102
60–69 years	215 (50.5)	260 (51.7)	15 (53.6)	19 (37.3)	–	294 (50.5)	
≥70 years	102 (23.9)	139 (27.6)	10 (35.7)	18 (35.3)	–	167 (28.7)	
Sex, *n* (%)							
Male	273 (64.1)	270 (53.7)	21 (75.0)	24 (47.1)	–	315 (54.1)	0.002
Female	153 (35.9)	233 (46.3)	7 (25.0)	27 (52.9)	–	267 (45.9)	
FIT							
Total CRC	164	32	3	6	–	41	
Age, *n* (%)							
50–59 years	28 (17.0)	5 (15.6)	1 (33.3)	4 (66.7)	–	10 (24.4)	0.003
60–69 years	88 (53.7)	10 (31.3)	0 (0)	0 (0)	–	10 (24.4)	
≥70 years	48 (29.3)	17 (53.1)	2 (66.7)	2 (33.3)	–	21 (51.2)	
Sex, *n* (%)							
Male	97 (59.2)	17 (53.1)	1 (33.3)	4 (66.7)	–	22 (53.7)	0.524
Female	67 (40.8)	15 (46.9)	2 (66.7)	2 (33.3)	–	19 (46.3)	

*CRC* colorectal cancer, *FIT* faecal immunochemical test, *gFOBT* guaiac-based faecal occult blood test.

^a^CRC cases in screening non-attendees were not specifically reported for gFOBT and FIT screening subgroups, as participants were not aware of which test would be offered when they had been invited for collecting a gFOBT/FIT kit from their general practitioners.

^b^*P* value refers to the difference between screen-detected and overall non-screen-detected CRCs.

### Tumour location and stage distribution

Tumour location and CRC stage distribution are described in Table 4. In the gFOBT-based screening programme, colonoscopy interval cancers (40.7%) were more commonly located in the proximal colon than screen-detected CRCs (28.7%), gFOBT interval cancers (32.6%) and CRCs among colonoscopy non-compliers (14.3%). While screen-detected CRCs (30.8%) and FIT interval cancers (26.9%) were less commonly found in the proximal colon, most colonoscopy interval cancers (66.7%) and CRCs in colonoscopy non-compliers (66.7%) were located proximally in the FIT screening subgroups. The stage distribution differed significantly among screen- and non-screen-detected groups, with more favourable stages in participants with screen-detected CRC (*P* < 0.001). Stage distribution was similar for gFOBT/FIT interval cancers and CRCs in non-attendees (*P* = 0.624). While a large proportion of early-stage screen-detected CRCs was observed in both gFOBT- and FIT-based screening programmes, the proportion of stage I cancers was much lower among CRCs detected by gFOBT than by FIT (39.3% vs. 49.3%).

**Table 4:** Tumour location and stage distribution of CRCs in the Wallonia–Brussels-organised gFOBT- and FIT-based screening programme.

	Screen-detected CRC	Non-screen-detected CRC					*P* value^a^
		Interval CRC		No colonoscopy compliance	CRC in non-attendees	Overall	
		gFOBT/FIT	Colonoscopy				
gFOBT + FIT							
Total CRC	590	535	31	57	13,973	14,596	
Tumour site, *n* (%)							
Proximal	159 (29.2)	162 (32.3)	13 (43.3)	11 (20.0)	3672 (28.6)	3858 (28.7)	0.788
Distal	385 (70.8)	339 (67.7)	17 (56.7)	44 (80.0)	9187 (71.4)	9587 (71.3)	
Stage, *n* (%)							
I	180 (40.9)	98 (23.8)	5 (25.0)	9 (19.1)	2164 (21.2)	2276 (21.3)	<0.001
II	122 (27.7)	106 (25.8)	5 (25.0)	9 (19.1)	2804 (27.5)	2924 (27.4)	
III	100 (22.7)	114 (27.7)	7 (35.0)	21 (44.7)	2889 (28.3)	3031 (28.4)	
IV	38 (8.6)	93 (22.6)	3 (15.0)	8 (17.0)	2339 (22.9)	2443 (22.9)	
gFOBT^b^							
Total CRC	426	503	28	51	–	582	
Tumour site, *n* (%)							
Proximal	119 (28.7)	155 (32.6)	11 (40.7)	7 (14.3)	–	173 (31.4)	0.375
Distal	295 (71.3)	320 (67.4)	16 (59.3)	42 (85.7)	–	378 (68.6)	
Stage, *n* (%)							
I	145 (39.3)	96 (23.7)	5 (25.0)	9 (19.6)	–	110 (23.4)	<0.001
II	102 (27.6)	106 (26.2)	5 (25.0)	8 (17.4)	–	119 (25.3)	
III	85 (23.0)	114 (28.1)	7 (35.0)	21 (45.7)	–	142 (30.1)	
IV	37 (10.0)	89 (22.0)	3 (15.0)	8 (17.4)	–	100 (21.2)	
FIT^c^							
Total CRC	164	32	3	6	–	41	
Tumour site, *n* (%)							
Proximal	40 (30.8)	7 (26.9)	2 (66.7)	4 (66.7)	–	13 (37.1)	0.474
Distal	90 (69.2)	19 (73.1)	1 (33.3)	2 (33.3)	–	22 (62.9)	
Stage, *n* (%)							
I	35 (49.3)	2 (33.3)	0 (0)	0 (0)	–	2 (28.6)	<0.001
II	20 (28.2)	0 (0)	0 (0)	1 (100)	–	1 (14.3)	
III	15 (21.1)	0 (0)	0 (0)	0 (0)	–	0 (0)	
IV	1 (1.4)	4 (66.7)	0 (0)	0 (0)	–	4 (57.1)	

*CRC* colorectal cancer, *FIT* faecal immunochemical test, *gFOBT* guaiac-based faecal occult blood test.

^a^*P* value refers to the difference between screen-detected and overall non-screen-detected CRCs.

^b^Unspecific location for screen-detected CRC, gFOBT interval cancer, colonoscopy interval cancer and CRCs in colonoscopy non-compliers are *n* = 12, 28, 1 and 2, respectively. Unspecific/missing stage for screen-detected CRC, gFOBT interval cancer, colonoscopy interval cancer and CRCs in colonoscopy non-compliers are *n* = 57, 98, 8 and 5, respectively.

^c^Unspecific location for screen-detected CRC and FIT interval cancer are *n* = 34 and 6, respectively. Unspecific/missing stage for screen-detected CRC, FIT interval cancer, colonoscopy interval cancer and CRCs in colonoscopy non-compliers are *n* = 93, 26, 3 and 5, respectively.

### Incidence rate of gFOBT/FIT interval cancer

Among 535 gFOBT/FIT interval cancers, 503 cases occurred during 236,102 person-years after a negative gFOBT and 32 cases occurred during 64,750 person-years after a negative FIT (Table 5). The incidence rates of gFOBT and FIT interval cancers were 21.3 (95% CI: 19.4–23.2) and 4.94 (95% CI: 3.23–6.65) per 10,000 person-years, respectively. Compared to participants with a negative gFOBT, those with a negative FIT were 77% less likely to develop an interval cancer (IRR 0.23, 95% CI: 0.16–0.33).

**Table 5:** Incidence rate of gFOBT and FIT interval cancer in the Wallonia–Brussels-organised screening programme.

Screening test	Interval cancer cases	Participants with negative gFOBT/FIT	Person-years	Incidence rate per 10,000 person-years (95% CI)	Incidence rate ratio (95% CI)
gFOBT	503	120,008	236,102	21.3 (19.4–23.2)	Reference
FIT	32	60,966	64,750	4.94 (3.23–6.65)	0.23 (0.16–0.33)

*CI* confidence interval, *FIT* Faecal immunochemical test, *gFOBT* guaiac-based faecal occult blood test.

## Discussion

In this population-based study, we compared important outcome variables of gFOBT- and FIT-based screening in the Wallonia–Brussels-organised CRC screening programme with comprehensive access to the dynamic data in regard to screening, insurance claims, pathology and cancer registration. This study showed that the detection rate and PPV for advanced neoplasms were approximately one third lower in gFOBT users compared to FIT users. Furthermore, the proportion and incidence rate of interval cancers were approximately four times higher with gFOBT than with FIT.

A major advantage of FIT is that it provides a quantitative result, which allows shifting the cut-off value of the test and adapting it to the specific needs of the population to be screened.^21–23^ A recent meta-analysis demonstrated that FIT sensitivity for detection of CRC increased from 69% at cut-offs 10–20 µg Hb/g to 80% at cut-offs ≤10 µg Hb/g.^24^ Consistently, sensitivity for detection of advanced adenomas increased from 21 to 31% at these cut-off values. While lower cut-off levels for referral to colonoscopy may be more effective when colonoscopy capacity is sufficient in screening programmes, higher cut-offs might be appropriate for programmes with limited colonoscopy resources. Furthermore, in contrast to gFOBT, FIT detects human Hb free of cross-reactivity with animal Hb and other dietary constituents. Additional advantages of FIT include the possibility of high-throughput automated processing as well as user-friendly application and higher adherence.^7,13,14,25,26^ Our results demonstrate that, at comparable positivity rates (6.0% for gFOBT vs. 7.7% for FIT), gFOBT is much less sensitive than FIT for clinically relevant colorectal neoplasms, which suggests that programmes switching from gFOBT to FIT will achieve better outcomes, even if using a FIT positivity threshold (at 15 µg Hb/g cut-off) that yields similar positivity rates as the gFOBT. Furthermore, given that the positivity rate of FIT among individuals previously screened with gFOBT (8.0%) was nearly the same as among FIT users without previous screening (7.7%), overall demands on colonoscopy capacity may not dramatically increase when switching to a FIT-based CRC screening programme.

In this study, there was no difference between gFOBTs and FITs concerning the higher detection rates among male participants. However, in contrast to gFOBT, the detection rates of advanced neoplasms strongly increased with age among FIT participants, suggesting that advantages of FIT-based screening over gFOBT-based screening are particularly large in the older age groups, in which prevalence and incidence of CRC are higher than in younger age groups.

Due to the lower sensitivity of gFOBT than FIT in detecting advanced neoplasia, interval cancer rates are expected to be lower in the latter screening programme. Studies from several European countries, including Spain, Denmark, France, Sweden and Scotland, have reported high proportions of interval cancer in the programmes offering gFOBT as an initial screening test (range: 48–58%).^27–31^ Reported proportions were much lower in studies from the Netherlands (23%),^32^ Slovenia (14%),^33^ and Italy (10%),^34^ where FIT is used as primary CRC screening test and positivity thresholds are set at levels 10–20 µg Hb/g faeces. Notably, with a high cut-off of 80 µg Hb/g faeces, the proportion of FIT interval cancer was remarkably similar to that seen with gFOBT.^35^ In this study, we demonstrated that the proportion of interval cancers dropped from 54.1% in gFOBT-based screening to 16.3% in FIT-based screening at the cut-off of 15 µg Hb/g faeces, with incidence rates of interval cancers falling from 21 to 5 cases per 10,000 person-years.

In the evaluation of gFOBT/FIT interval cancer rates, a minimum 2-year follow-up should be set up to observe all the potential interval cancers after a negative screening test.^36^ Although our study is based on a large number of person-years, we acknowledge the possibility of underestimation of gFOBT/FIT interval cancers due to insufficient follow-up time for those screened in the latter 2016 and in 2017. However, the proportion of FIT interval cancers observed in our study (16%) was very close to the proportion observed within the Flemish FIT-based screening programme (18%),^37^ which is another fully established organised screening programme in Belgium where FIT has been offered since October 2013, suggesting the potential underestimation to be small.

In the Wallonia–Brussels screening programme, most screen-detected CRCs, gFOBT/FIT interval cancers, CRCs in colonoscopy non-compliers and CRCs among non-attendees were located in the distal colon or rectum, whereas colonoscopy interval cancers tended to be located in the proximal colon. In our study, 31 colonoscopy interval cancers were detected and the majority (68%) occurred within 3 years after negative colonoscopy. Since colonoscopy interval cancers were more commonly found in the proximal colon, some procedural issues are needed to consider, such as inadequate bowel preparation and incompleteness of colonoscopy.^38–40^ While gFOBT/FIT interval cancer rates are related to test sensitivity, colonoscopy interval cancer may partly reflect colonoscopy quality. Both cancers should be carefully monitored when implementing a CRC screening programme.

In addition, 57 participants who refused to undergo colonoscopy after a positive gFOBT/FIT were later diagnosed with CRC. Unfortunately, no information on the reason for failing to use colonoscopy was available. To facilitate early detection of these cases, additional strategies to inform participants about the necessity of follow-up colonoscopy are needed. Interventions to reduce factors related to colonoscopy non-adherence, such as embarrassment, fear of the invasive procedure and examination result should be applied. Other outreach strategies could be built on sending all colonoscopy non-respondents a letter explaining the potential benefits or offering telephone calls to help them schedule colonoscopy appointments.

The success of a CRC screening programme not only depends on test diagnostic performance but to a very large extent also on adherence to screening invitation. The uptake rate was about 7% in the first year of the Wallonia–Brussels CRC screening programme implementation, whereas much higher participation rate (up to 50%) was achieved in the Flemish CRC screening programme.^37,41^ Several differences in screening strategies may explain this very large discrepancy. First, while the target population for screening were individuals aged 50–74 years in Wallonia and Brussels, people aged 56–74 years were invited for screening in Flanders. According to a prior study, the uptake rate of CRC screening was significantly lower in the age group 50–54 years compared to 55–59 years or older, but the difference was modest (48.0% vs. 55.6%).^42^ Second, the population in the Walloon and Brussels-capital region was initially invited merely by a letter without inclusion of a test kit, whereas the Flemish population was invited by an instruction letter along with a FIT kit. Third, in contrast to Flanders, there was no specific reminder for persons who did not collect a test kit from their GPs or who did not return the completed kit in Wallonia and Brussels. These major differences underline once more the overwhelming importance of designing practicalities of screening offers in a way that ensures maximum possible use and maximum possible effects. Even much higher uptake rates (71%) have been achieved in the Netherlands, where the success attributes to the implementation of real-time monitoring that allows immediate adjustments to the screening programme.^43^

To our knowledge, our study is the first to comprehensively evaluate the benefits of replacing gFOBT with FIT in a population-based CRC screening programme by comparing the detection rates, PPVs and incidence rates of gFOBT/FIT interval cancers. Our study population consists of average-risk individuals, comprising the age range commonly invited for CRC screening. Our findings may therefore be relevant for many countries considering implementation or modification of CRC screening programme. In addition, the large sample size enabled estimating key variables of gFOBT and FIT performance at high levels of precision.

Several limitations should be considered when interpreting our results. First, unexpected significant difference in the proportion of follow-up colonoscopy compliance was observed between individuals with positive gFOBT and those with FIT. Prior study also found similar variation and suggested the possible reason could be that gFOBT is a longer-standing screening test, the participants completing this test might be more likely to follow-up with subsequent testing.^7^ Notably, the detection rates of neoplasms were much lower among gFOBT users than FIT users, despite the higher completion rate of follow-up colonoscopy among gFOBT users. This implies that with higher colonoscopy adherence after positive FIT, even larger differences between gFOBT and FIT in detection rates may be observed. Second, as CRC progression may occur after the first months of a positive faecal test,^44^ a 2-year timeframe used to ascertain compliance rate of follow-up colonoscopy may have potentially inflated the number of screen-detected advanced neoplasms; however, given the almost unchanged PPVs for advanced neoplasm and proportion of screen-detected CRCs when considering follow-up colonoscopy as colonoscopy conducted within 6 months after positive faecal test, the impact of this prolonged follow-up window is likely to be limited. Third, colonoscopy information from the insurance claims database did not include important items such as size and number of detected polyps, or the quality of bowel preparation. Fourth, relatively low participation rates might reflect a relevant self-selection of participants, which might limit the generalisability of results to all screening programmes. Finally, we did not exclude FIT users having a history of gFOBT in the analysis on screen- and non-screen-detected CRCs, as we did not directly compare the characteristics of cancers and affected patients between the gFOBT- and FIT-based screening programmes. Also, this most likely reflects real-life conditions when a screening programme is in transition, which may provide better estimates than exclusively focusing on “pure” initial screening by FIT.

Despite these limitations, our study demonstrates that within a completely implemented organised CRC screening programme, replacing gFOBT with FIT strongly improved the advanced neoplasm detection rate and reduced interval cancer incidence. Implementation of FIT-based screening, along with measures to optimise screening adherence, is strongly encouraged in countries where gFOBT-based screening is still employed, or a new programme is in the process of planning.

## Supplementary information

Supplementary Apendix

Overview on the time periods covered by the various databases and used in our analysis

## Data Availability

Additional summary tables and sensitivity analyses are available upon reasonable request from the corresponding author.
