# Correction: Benefits of switching from guaiac-based faecal occult blood to faecal immunochemical testing: experience from the Wallonia–Brussels colorectal cancer screening programme

**DOI:** 10.1038/s41416-021-01468-w

**Published:** 2021-06-28

**Authors:** Feng Guo, Isabel De Brabander, Julie Francart, Michel Candeur, Marc Polus, Liesbet Van Eycken, Hermann Brenner

**Affiliations:** 1 Division of Clinical Epidemiology and Aging Research, German Cancer Research Center (DKFZ), Heidelberg, Germany; 2 Medical Faculty Heidelberg, University of Heidelberg, Heidelberg, Germany; 3 Belgian Cancer Registry (BCR), Brussels, Belgium; 4 Community Reference Center for Cancer Screening (Wallonia), Mont-Saint-Guibert, Belgium; 5 Department of Gastroenterology, University Hospital of Liège, Liège, Belgium; 6 Division of Preventive Oncology, German Cancer Research Center (DKFZ) and National Center for Tumor Diseases (NCT), Heidelberg, Germany; 7 German Cancer Consortium (DKTK), German Cancer Research Center (DKFZ), Heidelberg, Germany

**Keywords:** Health policy, Cancer screening, Cancer epidemiology

**Correction to:***British Journal of Cancer*10.1038/s41416-020-0754-5, published online 18 February 2020

The article benefits of switching from guaiac-based faecal occult blood to faecal immunochemical testing: experience from the Wallonia–Brussels colorectal cancer screening programme, written by Feng Guo, Isabel De Brabander, Julie Francart, Michel Candeur, Marc Polus, Liesbet Van Eycken and Hermann Brenner, was originally published electronically on the publisher’s internet portal on 18 February 2020 without open access. With the author(s)’ decision to opt for Open Choice the copyright of the article changed on 14th June 2021 to © The Author(s) 2021 and the article is forthwith distributed under a Creative Commons Attribution 4.0 International License, which permits use, sharing, adaptation, distribution and reproduction in any medium or format, as long as you give appropriate credit to the original author(s) and the source, provide a link to the Creative Commons licence, and indicate if changes were made. The images or other third party material in this article are included in the article’s Creative Commons licence, unless indicated otherwise in a credit line to the material. If material is not included in the article’s Creative Commons licence and your intended use is not permitted by statutory regulation or exceeds the permitted use, you will need to obtain permission directly from the copyright holder. To view a copy of this licence, visit http://creativecommons.org/licenses/by/4.0/ “http://creativecommons.org/licenses/by/4.0/”

Open Access funding enabled and organized by Projekt DEAL.

