# Supplementary material for: Benefits of switching from guaiac-based faecal occult blood to faecal immunochemical testing: experience from the Wallonia–Brussels colorectal cancer screening programme
Source: Br J Cancer. 2020 Feb 18;122(7):1109–17. doi: 10.1038/s41416-020-0754-5 (PMC7109124; doi:10.1038/s41416-020-0754-5)
Supplement: Supplementary file 1 — Supplementary Apendix [file 41416_2020_754_MOESM1_ESM.docx]

**Supplementary Appendix**

**Materials and Methods**

This section provides details on the sample collection and handling, laboratory analysis, quality management, and data handling according to the standard and check-list for faecal immunochemical tests for haemoglobin evaluation reporting (FITTER).^1^

*Sample collection and handling*

An automated quantitative faecal immunochemical test (FIT) (OC-Sensor, Eiken Chemical, Tokyo, Japan) was offered in the Wallonia-Brussels colorectal cancer (CRC) screening programme. The sample collection device for OC-Sensor consisted of a probe that holds approximately 10 mg of faeces and 2 ml of haemoglobin stabilization buffer. FIT participants were advised to stab the probe six times at random into one freshly passed whole faeces and then inserted back the probe into the device. Participants completed the sample collection and wrote the date of collection on the device label. Additionally, participants were asked to keep the collected samples in a refrigerator and return the samples to the Walloon Central Laboratory (Mont-Saint-Guibert, Belgium) as soon as possible.

*Laboratory analysis and quality management*

Returned samples were assayed on the day of receipt immediately using OC-Sensor io platform in the laboratory. Untreated samples during the day were stored at 4˚C and then allowed to warm to room temperature for analysis. The Walloon Central laboratory has a complete quality management system. Test calibrators and test controls were performed on a regular basis according to the manufacturer’ instructions. All analyses were carried out by laboratory-experienced staffs. The analytical working range was 10-200 µg haemoglobin per gram (Hb/g) faeces: specimens with faecal-Hb above the upper limit were not diluted and re-analyse.

*Data handling*

The faecal-Hb concentrations were recorded electronically by the analyser, along with the kit number, and these data were captured by the laboratory IT system. The general practitioners of the screening participants were informed on the test result within 5 days after analysis. Participants with faecal-Hb concentration > 15 µg Hb/g faeces (75 ng Hb/ml test buffer) were referred for diagnostic colonoscopy by their general practitioners.

**Supplementary Table 1**. Indicators of test performance of gFOBT versus FIT (colonoscopy conducted within 6 months after positive gFOBT/FIT)

|  | **Type of stool test used for screening** | | | | **Difference ^a^** |
| --- | --- | --- | --- | --- | --- |
|  | **gFOBT**  **Total N= 94 290** | | **FIT**  **Total N=28 217** | |  |
|  | **n** | **% (95% CI)** | **n** | **% (95% CI)** | **% (95% CI)** |
| **Positive gFOBT/FIT results** | 5645 | 6.0 (5.8-6.1) | 2185 | 7.7 (7.4-8.1) | 1.7 (1.4-2.1) |
| **Colonoscopy completion** | 4510 | 79.9 (78.8-80.9) | 1601 | 73.3 (71.4-75.1) | -6.6 (-8.8 to -4.5) |
| **Detection rate** |  |  |  |  |  |
| Colorectal cancer | 296 | 0.3 (0.3-0.3) | 116 | 0.4 (0.3-0.5) | 0.1 (0.0-0.2) |
| Advanced adenoma | 394 | 0.4 (0.4-0.5) | 246 | 0.9 (0.8-1.0) | 0.5 (0.3-0.6) |
| Any advanced neoplasm | 690 | 0.7 (0.7-0.8) | 362 | 1.3 (1.2-1.4) | 0.6 (0.4-0.7) |
| Non-advanced adenoma | 839 | 0.9 (0.8-0.9) | 544 | 1.9 (1.8-2.1) | 1.0 (0.9-1.2) |
| Any adenoma | 1233 | 1.3 (1.2-1.4) | 790 | 2.8 (2.6-3.0) | 1.5 (1.3-1.7) |
| **Positive predictive value ^b^** |  |  |  |  |  |
| Colorectal cancer | 296 | 6.6 (5.8-7.3) | 116 | 7.2 (6.0-8.5) | 0.6 (-0.8 to 2.1) |
| Any advanced neoplasm | 690 | 15.3 (14.2-16.4) | 362 | 22.6 (20.6-24.7) | 7.3 (5.0-9.6) |

CI, confidence interval; FIT, Faecal immunochemical test; gFOBT, guaiac-based faecal occult blood test

^a^ Differences with a 95% CI completely lower or higher than 0 are statistically significant, which means that the *P*-value does not exceed 0.05.

^b^ Positive predictive value is the percentage of participants with detected neoplasms relative to the number of participants with follow-up colonoscopy completed.

**Supplementary Table 2.** Characteristics of screen-detected CRC and affected patients (colonoscopy conducted within 6 months after positive gFOBT/FIT)

|  | **Type of stool test used for screening** | | |
| --- | --- | --- | --- |
|  | **gFOBT+FIT n (%)** | **gFOBT n (%)** | **FIT n (%)** |
| Total CRC | 567 | 408 | 159 |
| Age |  |  |  |
| 50-59 years | 132 (23.3) | 105 (25.7) | 27 (17.0) |
| 60-69 years | 291 (51.3) | 205 (50.2) | 86 (54.1) |
| ≥70 years | 144 (25.4) | 98 (24.0) | 46 (28.9) |
| Sex |  |  |  |
| Male | 357 (63.0) | 263 (64.5) | 94 (59.1) |
| Female | 210 (37.0) | 145 (35.5) | 65 (40.9) |
| Tumour site ^a^ |  |  |  |
| Proximal | 153 (29.3) | 115 (29.0) | 38 (30.2) |
| Distal | 369 (70.7) | 281 (71.0) | 88 (69.8) |
| Stage ^b^ |  |  |  |
| I | 177 (41.5) | 142 (40.0) | 35 (49.3) |
| II | 118 (27.7) | 98 (27.6) | 20 (28.2) |
| III | 95 (22.3) | 80 (22.5) | 15 (21.1) |
| IV | 36 (8.5) | 35 (9.9) | 1 (1.4) |

^a^ Unspecific locations for overall screen-detected CRC are 45.

^b^ Unspecific stages and unknown stages for overall screen-detected CRC are 50 and 91, respectively.

**Reference**

1. Fraser CG, Allison JE, Young GP, Halloran SP, Seaman HE. Improving the reporting of evaluations of faecal immunochemical tests for haemoglobin: the FITTER standard and checklist. *Eur J Cancer Prev* 2015; **24**(1): 24-26; e-pub ahead of print 2014/03/04; doi 10.1097/CEJ.0000000000000016.
