# Supplementary figures and images for: Benefits of switching from guaiac-based faecal occult blood to faecal immunochemical testing: experience from the Wallonia–Brussels colorectal cancer screening programme
Source: Br J Cancer. 2020 Feb 18;122(7):1109–17. doi: 10.1038/s41416-020-0754-5 (PMC7109124; doi:10.1038/s41416-020-0754-5)

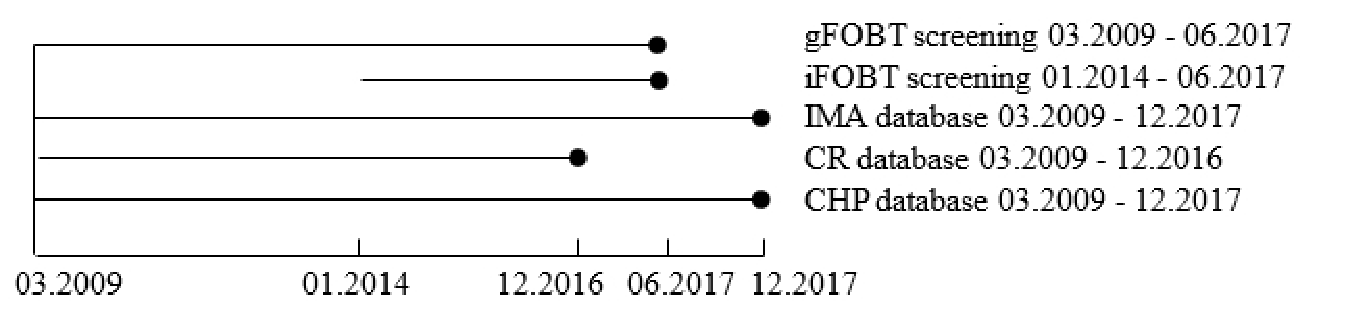

Supplement: Supplementary file 2 — Overview on the time periods covered by the various databases and used in our analysis [file 41416_2020_754_MOESM2_ESM.tif]
